# Protective Effects and Mechanisms of *Dendrobium nobile* Lindl. Alkaloids on PC12 Cell Damage Induced by A*β*_25-35_

**DOI:** 10.1155/2021/9990375

**Published:** 2021-08-16

**Authors:** Yuan Liu, Tingting Pi, Xiaohui Yang, Jingshan Shi

**Affiliations:** Department of Pharmacology and the Key Laboratory of Basic Pharmacology of Guhou Province, Zunyi Medical College, Zunyi, Guizhou Province, China 563000

## Abstract

**Background:**

A*β* deposition abnormally in the mitochondria can damage the mitochondrial respiratory chain and activate the mitochondrial-mediated apoptosis pathway, resulting in AD-like symptoms.

**Objective:**

To observe the protective effects of *Dendrobium nobile* Lindl. alkaloids (DNLA) on A*β*_25-35_-induced oxidative stress and apoptosis in PC12 cells explore its possible protective mechanisms.

**Methods:**

PC12 cells were treated with DNLA with different concentrations (0.035 mg/L, 0.3 mg/L, and 3.5 mg/L) for 6 h, followed by administration with A*β*_25-35_ (10 *μ*M) for 24 h. MTT assay and flow cytometer observe the effect of DNLA on A*β*_25-35_-induced cytotoxicity and apoptosis of PC12 cell. Based on the mitochondrial apoptosis pathway to study the antiapoptotic effect of DNLA on this model and its relationship with oxidative stress, flow cytometer detected the level of reactive oxygen species (ROS), and ELISA kits were used to detect superoxide dismutase activity (SOD) and glutathione (GSH) content in cells. The JC-1 fluorescent staining observed the effect of DNLA on the mitochondrial membrane potential (MMP) with inverted immunofluorescence microscopy. Western blot was used to detect the levels of mitochondrial apoptosis pathway-related protein and its major downstream proteins Bax, Bcl-2, cleaved-caspase-9, and cleaved-caspase-3.

**Results:**

DNLA can significantly improve the viability and apoptosis rate of PC12 cell damage induced by A*β*_25-35_. It also can restore the reduced intracellular ROS content and MMP, while SOD activity and GSH content increase significantly. The expression of apoptosis-related protein Bax, cleaved-caspase-9, and cleaved-caspase-3 decreased when the Bcl-2 protein expression was significantly increased.

**Conclusion:**

These findings suggest that it can significantly inhibit the apoptosis of PC12 cell damage induced by A*β*_25-35_. The mechanism may reduce the level of cellular oxidative stress and thus inhibit the mitochondrial-mediated apoptosis pathway.

## 1. Introduction

Alzheimer's disease (AD) is the most common type of dementia, accounting for 60%-70% of the overall incidence of dementia [1]. It is a severe neurodegenerative disease caused by the degeneration and loss of neurons in the brain [2]. AD pathological features are senile plaque (SP) caused by abnormal deposition of *β*-amyloid (A*β*) outside the cell and neurofibrillary tangles (NFTs) caused by hyperphosphorylation of tau protein inside the cell [3, 4]. However, the pathogeny and mechanism of AD have not yet been fully clarified, and the A*β* cascade hypothesis is currently widely accepted in many hypotheses about the onset of AD. A*β* is a common pathway for many factors that induce AD and a critical factor in the pathogenesis and development of AD [5, 6]. Studies have found that A*β*_1-40_ and A*β*_1-42_ are the main polypeptides that form SP, which are produced by the amyloid precursor protein (APP) under the cocutting action of *β*-secretase and *γ*-secretase [7–9]. However, A*β*_1-42_ is more hydrophobic than A*β*_1-40_ and more accessible to aggregate, leading to the formation of senile plaques (SP) [10]. The neurotoxic effects of A*β* are mainly related to mitochondrial-mediated apoptosis, disturbance of intracellular calcium homeostasis, and abnormal metabolism of reactive oxygen species (ROS) [10, 11]. A*β*_25-35_ is a synthetic fragment containing the recognized A*β* toxic amino acid sequence often used as a model drug in experiments. Therefore, it can be incubated with traditional methods to cause fiber aggregation to simulate the AD model [12, 13].

AD, as a neurodegenerative disease, is characterized by long-term neuronal death. Cell death mainly includes two forms of necrosis and apoptosis [14, 15]. Mitochondria are the place for energy metabolism and oxidative stress in nerve cells, which mediated apoptosis is the way for internal cell signals to trigger apoptosis [16]. Therefore, mitochondria-mediated apoptosis is likely to be an important part of the formation of AD. Spuch [17] found that the production of ROS and expression of Bax increased, while the expression level of Bcl-2 decreased in the AD model induced by A*β*. A*β* can damage the mitochondrial respiratory chain and increase ROS production to activate the mitochondrial-mediated apoptosis pathway, resulting in neuronal function decline and neuronal death. It can also cause AD symptoms such as memory loss, mental disorders, and movement disorders through the corresponding cascade reaction [18, 19]. Hence, A*β* plays a vital role in the early onset of AD, which is of great significance to use A*β* as the target to find effective prevention and treatment measures in the early onset of AD [20]. A*β* plays an important role and essential to find effective prevention and control measures as a target in the early stage of AD.

*Dendrobium nobile* Lindl. (DNL) is a precious Chinese medicinal material that was first published in “Shen Nong Materia Medica” and recorded in “Compendium of Materia Medica,” which has the functions of nourishing the stomach and kidney, strengthening brain and eyesight, and nourishing yin and lungs. The main components of DNL are alkaloids, polysaccharides, amino acids, sesquiterpenes, phenols, volatile oils, etc. among which *Dendrobium nobile* Lindl. alkaloids (DNLA) are the main active ingredients. Previous animal studies found that DNLA can effectively improve the learning and memory function of APP/PS1 transgenic mice, which also protect the neuron and prominent loss of A*β*_25–35_ induced model by promoting the expression of neurotrophic factors [21]. We also found that DNLA has a neuroprotective effect that can improve A*β*_25–35_ caused neuronal damage and reduces amyloid protein aggregation by inducing autophagy [22, 23]. Therefore, the use of A*β*_25–35_-induced PC12 cells to establish an AD model is to study the protection mechanism of DNLA.

## 2. Materials and Methods

### 2.1. Materials

Dendrobium was purchased from Xintian Traditional Chinese Medicine Industry Development Co., Ltd., of Guizhou Province. DNLA was isolated from the extracts and analyzed by LC-MS/MS. Alkaloids accounted for 79.8% of the DNLA and were comprised of 92.6% dendrobine (C16H25O2N), 3.3% dendrobine-N-oxide (C16H25O3N), 2.0% nobilonine (C17H27O3N), 0.9% dendroxine (C17H25O3N), 0.32% 6-hydroxy-nobilonine (C17H27O4N), and 0.07% 13-hydroxy-14-oxodendrobine (C16H23O4N) [21, 24]. A*β*_25–35_ was purchased from Sigma (American). FBS was purchased from EnmoAsay Biotechnology Co., Ltd. (China). Trypsin and DMEM/HIGHGLUCE medium were purchased from HyClone (America). MTT and DMSO were purchased from Solarbio (China). Mitochondrial membrane potential (MMP) detection and reactive oxygen detection kits were purchased from Biyuntian Biotechnology Research Institute (China). Anti-*β*-actin antibody, anti-GAPDH antibody, anti-Bcl-2 antibody, anti-Bax antibody, anti-caspase-3 (active) antibody, anti-caspase-3 antibody, anti-caspase-9 (active) antibody, and anti-caspase-9 antibody were purchased from Sangon Biotech (China). Biotin-conjugated Affinipure Goat Anti-Mouse IgG and Goat Anti-Rabbit IgG were purchased from Proteintech Group (China). Superoxide dismutase activity (SOD) and glutathione (GSH) content ELISA kit were purchased from Nanjing Jiancheng Bioengineering Institute (China). Annexin V-FITC/PI kit was purchased from Qihai Futai Biotechnology Co., Ltd. (China).

### 2.2. Cells

The differentiated PC12 cells were given to the Key Laboratory of the Department of Basic Pharmacology of Zunyi Medical College, which is derived from the adrenal pheochromocytoma in the brown house mouse and belongs to the sympathetic nervous system tumor.

### 2.3. Pretreatment of DNLA and A*β*_25-35_

DNLA was soluble in dimethyl sulfoxide (DMSO) and was stored at −20°C. When used, DNLA was diluted to different concentrations with DMEM/HIGHGLUCE medium containing 10% fetal bovine serum and 1% penicillin-streptomycin mixture.

Add sterile PBS of 943.85 *μ*L to 1 mg of A*β*_25-35_ to fully dissolve it to make the concentration 1 mM. Put it in a 37°C incubator and incubate for 7 days. The working solution diluted to 100 *μ*mol/L is added to the culture medium to make the final concentration 10 *μ*mol/L when used.

### 2.4. Cell Culture of PC12

Used DMEM/HIGHGLUCE medium containing 10% fetal bovine serum and 1% penicillin-streptomycin mixture for PC12 cell culture, planted in 25 cm^2^ cell culture flask, and placed in 37°C with 5% CO2 constant temperature incubator. The cells were subcultured or seeded when they grew to 80% in the bottom of the bottle. When passaging, the culture medium should be discarded first, washed once with autoclaved PBS, and discarded. After digestion with 0.25% trypsin for 2 min, stop the digestion with serum-containing culture medium and blow the cells into the culture medium. Centrifuge at 1000 rpm for 5 min, discard the supernatant, and add serum-containing medium to resuspend and inoculate in a cell culture flask.

### 2.5. Groups and Administration

PC12 cells were divided into five groups and different concentrations of DNLA after 24 h cell seeding: control group, model group (10 *μ*M A*β*_25–35_), DNLA low-dose group (DNLA-L, 0.035 mg/L DNLA+10 *μ*M A*β*_25–35_), DNLA medium-dose group (DNLA-M, 0.35 mg/L DNLA+10 *μ*M A*β*_25–35_), and DNLA high-dose group (DNLA-H, 3.5 mg/L DNLA+10 *μ*M A*β*_25–35_). A*β*_25–35_ was given 6 h later, and the relevant indicators were tested after 24 h.

### 2.6. Assessment of Cell Viability by MTT Assays

Neurons were seeded into 96-well plates and treated with DNLA for 30 h at different concentrations. After five replicates were made for each treatment, cell viability was evaluated by MTT assays as previously described [24]. The cell viability is expressed as an OD percentage of cells with the indicated treatments in cells treated with the DMSO control treatment.

### 2.7. Assessment of Cell Apoptosis by Flow Cytometer

The PC12 cell apoptosis assay was performed using the Annexin V-FITC/PI kit following the manufacturer's protocol. Transfer the medium to the centrifuge tube separately after each group of A*β*_25-35_ acts for 24 h. In the culture plate, add 200 *μ*L of trypsin (without EDTA) to each well to digest the cells. After the digestion is complete, add the original medium back to the corresponding culture well to terminate the digestion. Transfer the cells to a centrifuge tube and centrifuge at 1400 rpm for 4 minutes, then aspirate the medium. Resuspend in PBS and centrifuge at 1400 rpm for 4 min and discard the supernatant twice. Resuspend the cells with 400 *μ*L 1× binding buffer and add 5 *μ*L Annexin V-FITC to mix well, then incubate for 15 minutes at room temperature in the dark. Add 10 *μ*L of PI staining solution into each tube to mix well and incubate for 5 minutes on ice (2°C to 8°C) in the dark. The mixtures were incubated for 10 min. The samples were then filtered through a nylon mesh into a tube. The samples were tested by using the flow cytometer.

### 2.8. Assessment of ROS Level by Flow Cytometer

The total ROS was measured by flow cytometry according to the ROS kit manufacturer's protocol. DCFH-DA in the kit is diluted 1 : 1000 with a serum-free medium, and the concentration of the working solution is 10 *μ*M. Use A*β*_25–35_ for 24 h after administration according to groups, wash away the original medium, and wash once with sterile PBS. Add 1 mL of working solution to each well of the culture plate, incubate in the cell incubator for 30 minutes, and shake every 10 minutes. Wash three times with serum-free medium and add 0.25% trypsin to digest and stop digestion by adding medium. All the samples were centrifuged at 1400 rpm for 4 min, and the supernatants were resuspended in PBS. The fluorescent intensities were measured using a flow cytometer.

### 2.9. Assessment of SOD Activity and GSH Level by ELISA Kit

The cells were digested with trypsin and added the original medium to terminate the digestion. The cell was centrifuge at 1400 rpm for 4 min and aspirated the supernatant to add PBS to resuspend the cells three times. The samples were disrupted by ultrasound for four times (6 s each time), 1 min interval each time. The samples were tested by using the SOD and GSH content kit following the manufacturer's protocol.

### 2.10. Assessment of MMP by JC-1 Fluorescent Staining

Aspirate and discard the original medium of the cells to be tested, add sterile PBS, and wash once. The preprepared working solution was added (1 : 1) to the serum-free medium and incubated for 30 min in the cell incubator. The plate's medium was discarded and washed twice with a diluted JC-1 staining buffer (1×). The sample was added serum-free medium and observed with an inverted fluorescence microscope.

### 2.11. Western Blot Assay

Total protein was extracted from cultured neurons using a total protein extraction kit and quantified by a BCA protein assay kit. Equal amounts of protein (20 mg) per lane were separated by Nu-PAGE gels and then transferred to a PVDF (0.45 mm) membrane. The membranes were incubated with the following primary antibodies: anti-Bax (1 : 1000), anti-Bcl2 (1 : 1000), anti-caspase-9 (1 : 1000), anti-caspase-3 (1 : 1000), anti-GAPDH (1 : 2000), and anti-*β*-actin (1 : 2000) at 4°C overnight, followed by incubation with secondary antibody at 4°C for 1 h. The membranes were visualized using the chemiluminescence reagent ECL Plus (E003-100). The image was scanned, and band densities were quantified using Quantity One 1D analysis software v4.52 (Bio-Rad).

### 2.12. Statistical Analysis

All data were presented as mean ± standard error. Data were analyzed by SPSS 19.0 statistics software. The data were analyzed by one-way ANOVA, and the statistical significance of the difference between the two groups was determined using the LSD method if the equal variance or Dunnett's T3 method has a missing variance. *P* < 0.05 was considered statistically significant.

## 3. Result

### 3.1. Effects of DNLA on the Cell Viability of PC12 Cells

**Figure 1:**
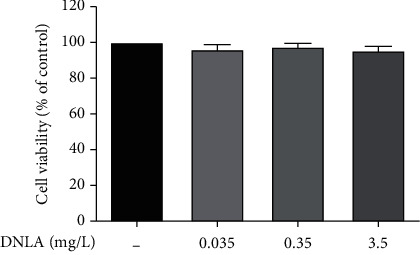
Effect of DNLA on cell viability in PC12 cells. The cell viability in PC12 cells after treatment with DNLA (*n* = 6, $\bar{x}\pm s$).

In this experiment, PC12 cells were administered with different concentrations of DNLA for 30 h, and the MTT assay was used to detect the cell viability. The results showed no significant change in the cell viability after 30 h of DNLA at different concentrations (Figure 1, *P* > 0.05), which indicated the concentration of DNLA used in this experiment has no toxic effect on PC12 cells.

### 3.2. Effect of DNLA on the Viability of PC12 Cells Induced by A*β*_25–35_

**Figure 2:**
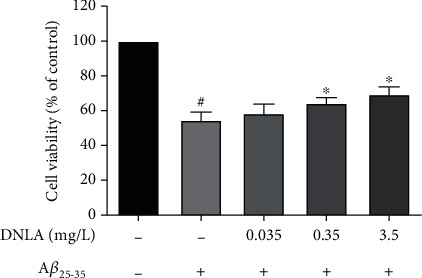
Effect of DNLA on cell viability induced by A*β*_25-35_ in PC12 cells. The cell viability in PC12 cell damage induced by A*β*_25-35_ after treatment with DNLA (*n* = 6, $\bar{x}\pm s$). Note: ^#^*P* < 0.05 vs. control; ^∗^*P* < 0.05 vs. model.

The MTT assay showed that after 24 h of induction by A*β*_25–35_ (10 *μ*M),and the cell viability was significantly lower than the control group. Cells were given different concentrations of DNLA for 6 h in advance that can increase the cell viability of PC12 cells damage induced by A*β*_25–35_ in a dose-dependent manner. The cell viability of 0.35 mg/L and 3.5 mg/L in the DNLA dose groups was significantly higher than the model group (Figure 2, *P* < 0.05).

### 3.3. Effect of DNLA on PC12 Cell Apoptosis Induced by A*β*_25-35_

**Figure 3:**
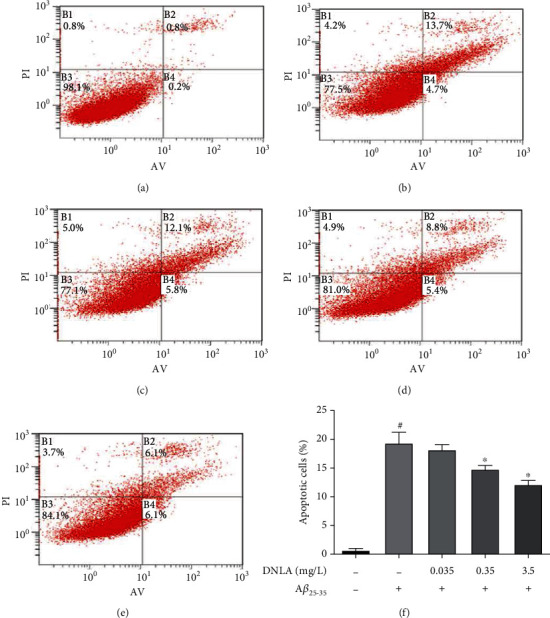
Effect of DNLA on PC12 cell apoptosis induced by A*β*_25-35_. (a–e) Flow cytometry detection of Annexin V-FITC/PI staining to detect apoptotic cells in control, model, DNLA-L, DNLA-M, and DNLA-H groups, respectively. (f) The number of the apoptosis cells (*n* = 3, $\bar{x}\pm s$). Note: #*P* < 0.05 vs. control; ^∗^*P* < 0.05 vs. model.

In this experiment, the Annexin V/PI staining method was used to detect each group of cells by flow cytometry. The results showed (Figure 3(a), *P* < 0.05) that the apoptosis rate of the model group given A*β*_25–35_ was significantly higher than the control group, and the level of apoptosis induced by A*β*_25-35_ could be reduced by giving DNLA in advance, which was concentration-dependent. The statistical results showed that the 0.35 mg/L and 3.5 mg/L DNLA dose groups could significantly inhibit A*β*_25-35_-induced apoptosis, which difference is statistically significant as compared with the model group (Figure 3(b), *P* < 0.05).

### 3.4. Effect of DNLA on the Level of ROS in PC12 Cell Damage Induced by A*β*_25-35_

**Figure 4:**
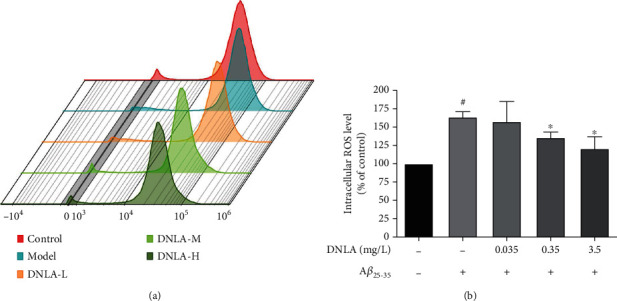
Effect of DNLA on the level of ROS in PC12 cell damage induced by A*β*_25-35_. (a) Flow cytometry detection of DCFH-DA staining to detect intracellular ROS level in control, model, DNLA-L, DNLA-M, and DNLA-H groups, respectively; (f) The intracellular ROS level of the cells (*n* = 3, $\bar{x}\pm s$). Note: #*P* < 0.05 vs. control; ^∗^*P* < 0.05 vs. model.

The flow cytometry results (Figure 4(a), *P* < 0.05) showed that the absorption peak of the model group that was given to A*β*_25-35_ was significantly shifted to the right compared with the control group. The absorption peak was shifted to the left compared with the model group by giving DNLA in advance, which was concentration-dependent. The statistical results showed (Figure 4(b), *P* < 0.05) that the administration of A*β*_25-35_ can significantly increase the intracellular ROS levels. After the administration of DNLA, it can effectively inhibit the production of intracellular ROS and gradually decrease the ROS level with the dose increase. DNLA at the doses of 0.35 mg/L and 3.5 mg/L can significantly inhibit the increase in ROS levels caused by A*β*_25-35_ when compared with the model group, which difference is statistically significant (Figure 4(b), *P* < 0.05).

### 3.5. Effect of DNLA on the GSH Content and SOD Activity of PC12 Cell Damage Induced by A*β*_25-35_

**Figure 5:**
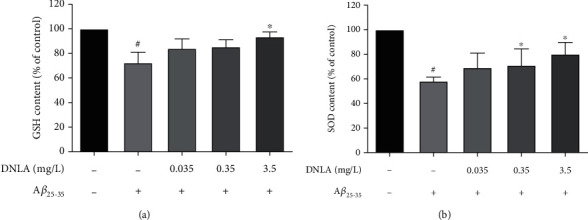
Effect of DNLA on the GSH content and SOD activity of PC12 cell damage induced by A*β*_25-35_. (a) The intracellular GSH level in PC12 cells. (b) The intracellular SOD activity in PC12 cells (*n* = 3, $\bar{x}\pm s$). Note: ^#^*P* < 0.05 vs. control; ^∗^*P* < 0.05 vs. model.

The endogenous nonenzymatic antioxidant GSH and antioxidant enzyme SOD can effectively remove oxygen free radicals in the body, which content and activity can indirectly reflect the oxidative stress state of cells. The results showed that the GSH content and SOD activity of the model group were significantly reduced when compared with the control group (Figures 5(a) and 5(b); *P* < 0.05). After administering DNLA in advance, the intracellular GSH content of the 3.5 mg/L DNLA dose group and the SOD activity of the 0.35 mg/L and 3.5 mg/L DNLA dose groups were effectively improved as compared with the model group (Figures 5(a) and 5(b); *P* < 0.05).

### 3.6. Effect of DNLA on A*β*_25-35_-Induced Mitochondrial Apoptosis Pathway Proteins in PC12 Cells

**Figure 6:**
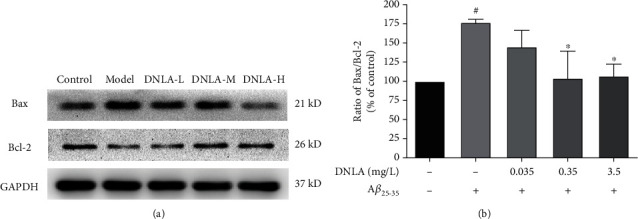
Effect of DNLA on the expression of Bax and Bcl-2 induced by A*β*_25-35_ in cells. (a) Respective photomicrographs of A*β*_25-35_-induced mitochondrial apoptosis pathway proteins. (b) The ratio of Bax/Bcl-2 in PC12 cells (*n* = 3, $\bar{x}\pm s$). Note: ^#^*P* < 0.05 vs. control; ^∗^*P* < 0.05 vs. model.

This experiment observed the effect of DNLA on the expression of Bax and Bcl-2 proteins in PC12 cell damage induced by A*β*_25-35_. The statistical results showed that (Figure 6(b), *P* < 0.05) the expression level of Bax protein increased, and the expression level of Bcl-2 protein decreased in the model group when compared with the control group. Early administration of DNLA can inhibit the upregulation of A*β*_25-35_ on Bax protein and the downregulation of Bcl-2 protein levels when compared with the control group. Through the statistics of the ratio of the gray value of Bax and Bcl-2 protein bands, the ratio of Bax/Bcl-2 in the model group was significantly increased compared with the control group. Compared with the model group, the Bax/Bcl-2 ratios of 0.35 mg/L and 3.5 mg/L DNLA dose groups are significantly reduced, which difference is statistically significant (Figure 6(b), *P* < 0.05).

### 3.7. Effect of DNLA on the MMP of PC12 Cell Damage Induced by A*β*_25-35_

**Figure 7:**
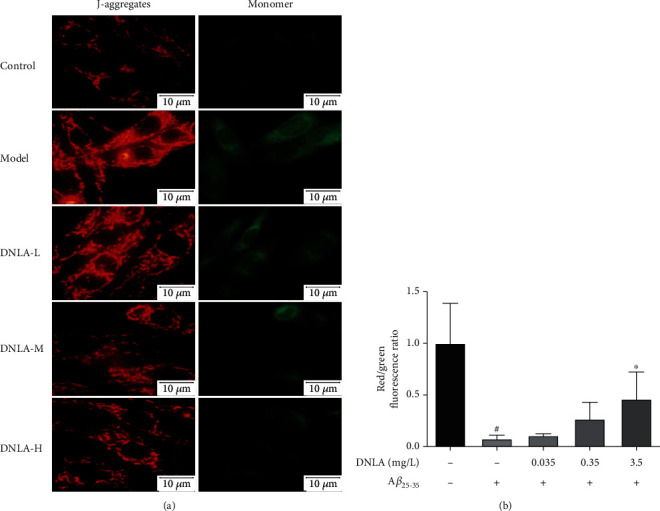
Effect of DNLA on MMP induced by A*β*_25-35_ in PC12 cells. (a) showed the respective photomicro in PC12 cells. (b) The ratio of red/green fluorescence in PC12 cells (*n* = 5, $\bar{x}\pm s$). Note: ^#^*P* < 0.05 vs. control; ^∗^*P* < 0.05 vs. model.

Compared with the control group, JC-1 fluorescent staining results showed that the model group shows the ratio of red/green fluorescence, and fluorescence is significantly decreased (Figure 7(b), *P* < 0.05). The ratio of red/green fluorescence increased in the DNLA administration group as the dose increased when compared with the model group (Figure 7(b), *P* < 0.05). The results showed that A*β*_25-35_ could induce a decrease in MMP. The administration of DNLA in advance can effectively inhibit the decrease of MMP caused by A*β*_25-35_.

### 3.8. Effect of DNLA on Caspase-9 and Caspase-3 in PC12 Cell Damage Induced by A*β*_25-35_

**Figure 8:**
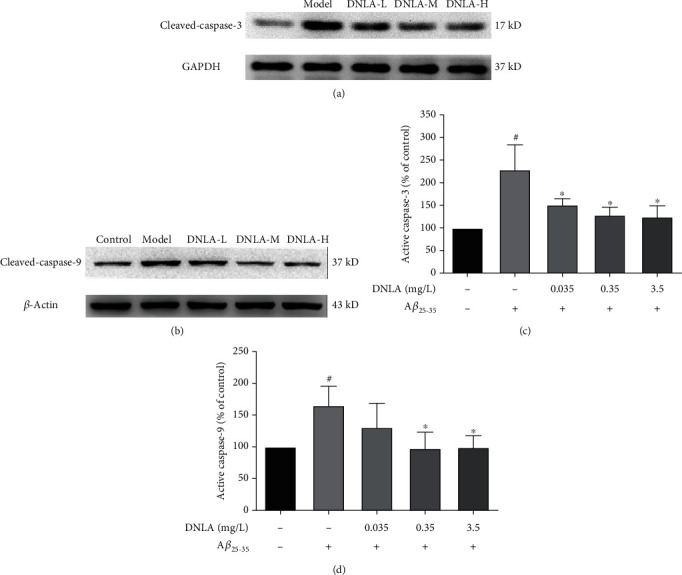
Effect of DNLA on the expression of cleaved-caspase-9 and cleaved-caspase-3induced by A*β*_25-35_ in PC12 cells. (a) Respective photomicrographs of cleaved-caspase-9 in PC12 cell damage induced by A*β*_25-35_. (b) Respective photomicrographs of cleaved-caspase-3 in PC12 cell damage induced by A*β*_25-35_. (c) The cleaved-caspase-9 activity in PC12 cells (*n* = 3, $\bar{x}\pm s$). (d) The cleaved-caspase-3 activity in PC12 cells (*n* = 3, $\bar{x}\pm s$). Note: ^#^*P* < 0.05 vs. control; ^∗^*P* < 0.05 vs. model.

Compared with the control group, western blot analysis showed that the protein expression levels of cleaved-caspase-9 and cleaved-caspase-3 in the model group were significantly increased (Figures 8(c) and 8(d); *P* < 0.05). However, after administration of 0.35 mg/L and 3.5 mg/L DNLA dose groups can effectively reduce the protein expression levels of cleaved-caspase9 and cleaved-caspase-3, difference is statistically significant (Figures 8(c) and 8(d); *P* < 0.05). Therefore, DNLA can effectively inhibit the activation of caspase-9 and caspase-3 in PC12 cell damage induced by A*β*_25-35_.

## 4. Dissussion

This study found that DNLA has a protective effect on PC12 cell damage induced by A*β*_25-35_, which reduced the rate of cell apoptosis and improved the oxidative stress level of cells by protecting mitochondria. Preadministration of DNLA can inhibit ROS generation and improve the oxidative stress state of the PC12 cell damage induced by A*β*_25-35_. In addition, the expression of mitochondrial-mediated apoptosis pathway-related proteins Bax, cleaved-caspase9, and cleaved-caspase3 in the administration group was significantly lower than the model group, whereas the antiapoptotic protein Bcl-2 was significantly higher than the model group. The MMP was significantly improved, which effectively inhibited the apoptosis rate of cells. These results suggested that DNLA has a protective effect on apoptosis and oxidative stress for A*β*_25-35_-induced cells.

DLNA was given in advance to explore its possible mechanism of action. Since it is sometimes impossible to determine the impact of a drug at a concentration, we used the MTT experiment to determine the cell survival rate to determine the effective range. In this study, we used three concentrations within the effective range to observe whether the drug is dose-dependent [22, 24], and the MTT experiment was used to observe the cytotoxicity of A*β*_25-35_ to PC12 cells. The results showed that the cell viability of the MTT experiment in the model group decreased significantly, suggesting A*β*_25-35_ can produce cytotoxicity to PC12 cells and successfully established as the experimental model. However, the cell viability of 0.35 mg/L and 3.5 mg/L DNLA dose groups was significantly higher than the model group in the MTT assays, which suggests DNLA inhibited the cytotoxicity induced by A*β*_25-35_. The experimental results found that the 0.035 mg/L DNLA dose group did not reduce the cytotoxicity, indicating that the 0.035 mg/L DNLA dose group did not reach the threshold dosage under the experimental conditions.

Abnormal deposition of A*β* can trigger neuronal apoptosis and participate in the formation of AD as the main cause of neuronal loss [25, 26]. In this study, the Annexin V/PI double staining method was used to detect the apoptosis of PC12 cells by flow cytometry to explore the reasons why A*β*_25-35_ induced PC12 cells to produce cytotoxicity. Annexin V binds with high affinity and specificity to phosphatidylserine that appears on the cell membrane surface during apoptosis. At the same time, PI can stain cells that lose cell membrane integrity in the late stage of apoptosis [25]. The results showed that the apoptosis rate of PC12 cell damage induced by A*β*_25-35_ increased significantly, suggesting that A*β*_25-35_ caused PC12 cell apoptosis is consistent with a previous study [26]. The apoptotic levels of 0.35 mg/L and 3.5 mg/L DNLA dose groups was significantly lower than that of the model group, but the apoptosis level of the 0.035 mg/L DNLA dose group did not significantly improvement, which indicated that the effective dose of DNLA can inhibit A*β*_25-35_-induced apoptosis under this experimental conditions. Similarly, previous studies have also shown that DNLA can improve A*β*_25-35_-induced axonal degeneration of hippocampal neurons in rats and ameliorate ER dilation and swelling in the hippocampal neurons [22, 27, 28].

Oxidative stress is the mediator of apoptosis caused by A*β* [29, 30]. Oxidative stress refers to the imbalance between oxidation and antioxidation in the body that causes excessive ROS production in the body, resulting in cell and tissue damage [31, 32]. As the main indicator of oxidative stress level, ROS can damage nucleic acids and proteins by attacking fatty acids on cell membranes to produce peroxides, triggering inflammation and apoptosis to accelerate aging. It is one of the leading causes of chronic diseases, such as heart disease and AD [33, 34]. Studies have shown that the degree of oxidation of DNA and protein in the brain tissue of AD patients is significantly increased, indicating that oxidative stress may accelerate the formation of AD by accelerating cognitive dysfunction and pathological brain damage in AD patients [35, 36]. Relevant animal and cell experiments also showed that abnormally deposited A*β* can interfere with reduced coenzyme I (NADH) through A*β*-binding alcohol dehydrogenase (ABAD) in the mitochondria to affect the respiratory chain and increase ROS production, aggravating A*β* deposition and mitochondrial dysfunction to accelerate the formation of AD [37–39].

Therefore, the DCFH-DA method was used to detect the intracellular ROS level [40]. DCFH-DA can enter the cell through the cell membrane and be hydrolyzed into DCFH by esterase which further react with intracellular ROS and oxidize into fluorescent DCF [41]. The intracellular ROS level can be judged by detecting the intensity of fluorescence. Our experimental results found that the intracellular ROS level of the model group was significantly higher than that of the control group, which is consistent with reports in the literature [42]. However, the intracellular ROS levels in the DNLA 0.35 mg/L and 3.5 mg/L DNLA dose groups were significantly lower than the model group. Furthermore, this experiment detected the main SOD activity and GSH content to observe the oxidative stress level of cells. The results showed that the intracellular SOD activity and GSH content of the model group induced by A*β*_25-35_ were significantly lower than that of the control group, but the effective dose of DNLA could significantly increase the SOD activity and GSH content after the intervention. The above results suggested that the effective dose of DNLA under the experimental conditions can reduce the increase in intracellular ROS levels induced by A*β*_25-35_, and it also can inhibit the oxidative stress level of cells by upregulating SOD activity and GSH content.

Mitochondria are not only the site of oxidative stress but also the organelle for energy production in the cell. Its function is of great significance for maintaining the stability of neuronal function and the integrity of synapses [43]. Moreover, the inner mitochondrial membrane is the processing site of mitochondrial enzymes that indirectly reflects mitochondria's function [44, 45]. The results of our previous in vitro experiments showed that DNLA could inhibit the reduction of MMP caused by oxidative stress and has a positive protective effect on the mitochondrial function [46]. Therefore, JC-1 fluorescent staining was used to observe the protective effect of DNLA on the mitochondrial membrane of PC12 cells damage induced by A*β*_25-35_. JC-1 can quickly enter the mitochondria when the MMP is high and exist stably in the mitochondrial matrix in the form of a red fluorescent polymer. JC-1 can escape the mitochondria and exist as a green fluorescent monomer while the MMP decreases. JC-1 fluorescent staining observed that the red fluorescence of the model group was diffusely distributed than that of the control group, and the green fluorescence was strongly expressed. It was consistent with previous study [22]. However, early intervention of an effective dose of DNLA could reduce cell diffuse red fluorescence, and green fluorescence was concentration-dependent. The results showed that A*β*_25-35_ could induce a decrease of intracellular MMP and destroy the integrity of the mitochondrial membrane, which is consistent with the results of previous in vitro studies [21, 47]. After intervention with DNLA, it can significantly inhibit the decrease of MMP and has a positive protective effect on mitochondrial function. It is suggested that DNLA has a protective effect on MMP, and it may be the result of improving the level of oxidative stress by inhibiting ROS production.

The mitochondrial-mediated apoptosis pathway involved in reduction of MMP [48, 49] is an apoptotic mode triggered by Bcl-2 family proteins [50]. Bax mainly controls the integrity of the outer mitochondrial membrane and forms a homodimer with itself to control the permeability of the mitochondrial membrane when overexpressed [51, 52]. It releases cytochrome C into the cytoplasm to activate the core components of cysteine and aspartic proteases, caspase-9 and caspase-3, to complete the mitochondrial-mediated apoptosis pathway, leading to cell apoptosis [53, 54]. As an antiapoptotic protein, Bcl-2 can form a heterodimer with Bax to prevent the release of cytochrome C to achieve the purpose of inhibiting apoptosis [55, 56]. Therefore, the composition ratio of Bcl-2 family members is a key factor in regulating apoptosis; especially, the balance of Bax/Bcl-2 in cells plays an important role in the development of neuronal apoptosis and AD [57]. This experiment further explored the effect of DNLA on the expression of mitochondrial apoptosis pathway-related proteins in the PC12 cell model by western blotting. We found that the ratio of the Bax/Bcl-2 protein and the protein expression levels of cleaved-caspase-9 and cleaved-caspase-3 induced by A*β*_25-35_ in the model group were significantly upregulated compared to the control group cells. However, the ratio of the Bax/Bcl-2 protein was reduced after the administration of DNLA, and the protein expression of downstream cleaved-caspase-9 and cleaved-caspase-3 was downregulated. The results indicated that early intervention with effective doses of DNLA might reduce the level of intracellular oxidative stress and inhibit the mitochondrial-mediated apoptosis pathway, thereby decreasing the apoptosis induced by A*β*_25-35_ and achieving the purpose of protecting cells.

In conclusion, we found that administering an effective dose of DNLA in advance can significantly improve the apoptosis induced by A*β*_25-35_. The mechanism may be related to reducing ROS production and level of oxidative stress, which in turn inhibits the pathway of mitochondrial apoptosis. This experiment provides a basic pharmacological basis for DNLA to delay development of AD when combined with the results of the previous research.

## 5. Conclusion

Under the experimental conditions, an effective dose of DNLA can significantly inhibit the apoptosis of PC12 cell damage induced by A*β*_25-35_, which may reduce the level of cellular oxidative stress and inhibiting the mitochondrial-mediated apoptosis pathway.

## Data Availability

The data used to support the findings of this study are available from the corresponding author upon request.
